# Evaluation of Pulsed Alternating Wavelength System Lighting on the Welfare Quality and Serotonin Turnover of Commercial Laying Hens Throughout a Lay Cycle

**DOI:** 10.3390/ani16020241

**Published:** 2026-01-13

**Authors:** Brittney J. Emmert, Sara Tonissen, Jenna M. Schober, Gregory S. Fraley, Darrin M. Karcher

**Affiliations:** Department of Animal Sciences, Purdue University, West Lafayette, IN 47907, USA; emmertb@purdue.edu (B.J.E.); stonisse@purdue.edu (S.T.); jschober@purdue.edu (J.M.S.); gfraley@purdue.edu (G.S.F.)

**Keywords:** lighting, laying hen, neurotransmitter, welfare, conventional cage

## Abstract

Laying hens require lighting for proper development and reproduction. There is limited research on the effects that lighting types have on birds’ welfare quality. A novel lighting source, Pulsed Alternating Wavelength System (PAWS), is being evaluated in the industry that claims to improve birds’ growth rate, decrease age at first egg, and decrease aggressive and nervous behaviors. Understanding how PAWS effects hen’s welfare, both physically and physiologically, is critical if this technology is to be adopted by industry. The project evaluated the effects of two PAWS lighting recipes on neurotransmitter turnover and welfare quality of commercial, conventionally caged laying hens. Three flocks of White leghorn hens (control [fluorescent lights] and two PAWS flocks [PAWS1 and PAWS2]) were sampled from 22 to 70 weeks of age, depending on the flock. The physical welfare of 50 hens per flock and neurotransmitter turnover of 10 hens per flock were assessed at each timepoint. The majority of welfare quality parameters were influenced by age as opposed to lighting type. No differences in dopamine turnover were observed. The hens housed under PAWS1 had reduced serotonin turnover, thus increased serotonin activity, and PAWS2 hens had improved keel bone damage scores; both indicative of improved welfare compared to control hens. The novel lighting may be beneficial to layer welfare, which may lead to increased longevity and productivity. Implementation in cage-free housing should be explored to delve into potential behavioral differences that could further influence welfare outcomes.

## Simple Summary

Artificial light sources are utilized in the laying hen industry to maintain proper growth and egg production. The Pulsed Alternating Wavelength System (PAWS) is a new lighting type that may benefit laying hen production and welfare, but needs to be validated. This study evaluated the effects on neurotransmitter turnover (physiological markers of welfare) and physical welfare quality of commercial laying hens housed in conventional cages under two PAWS recipes or fluorescent lights throughout a lay cycle. The majority of welfare parameters were influenced by age and not lighting type. However, one PAWS recipe had reduced serotonin turnover, and the other PAWS recipe had reduced keel bone damage. These are both indicators of improved physiological and physical welfare compared to the control hens. The PAWS lighting may be beneficial to the welfare of conventionally caged laying hens and warrants further research, especially in alternative housing systems.

## 1. Introduction

Livestock industries are rapidly adapting and adopting new technologies to improve the growth, production, and welfare of animals. One novel technology, the Pulsed Alternating Wavelength System (PAWS; XTi Lighting, Inc., Greeley, CO, USA), has been developed that delivers multiple wavelengths of light in an alternating fashion and may be beneficial to the growth and welfare of poultry. Over 3 million pullets and laying hens have been kept under PAWS lighting [1], which, according to personal communications with the developers, has accelerated pullets’ growth rate, decreased the age at first egg, and decreased aggressiveness and nervousness behaviors. Thus far, all hens under PAWS have been housed in conventional cages with no published information on the impact on laying hen production and welfare.

Previous research has established the effect of various lighting types on laying hen productivity. Single wavelength light can affect egg production, shell quality, and the time from first egg to 50% production [2,3,4]. There are no published effects of PAWS on the production, welfare (affective state), or skeletal system of laying hens. However, considering that other lighting parameters, such as wavelength, do have various effects on poultry, there is reason to hypothesize that PAWS will have an effect as well.

In addition to visually observing a hen’s physical welfare, assessing chemical regulators of their mental state can provide additional insight into welfare and well-being that cannot be outwardly seen. Neurotransmitters and neuromodulators are the active regulators of an animal’s mental state. Serotonin (5-HT) and dopamine (DA) are two neurotransmitters associated with affective state regulation [5,6,7]. Within the brain, there is a balance of excitatory and inhibitory signals that control cognition, emotion, and homeostasis [8]. These signals are created by the synthesis of neurotransmitters and neuromodulators in the presynaptic neuron, their release into the synapse, and subsequent interaction with their receptors on the postsynaptic neuron. The activation of these receptors creates a signal cascade that may lead to the alteration of brain signaling and mental state. However, instead of receptor activation, neurotransmitters can be inactivated by reuptake back into the presynaptic neuron and degraded into metabolites. Therefore, 5-HT and DA turnover should be evaluated in addition to the static levels to correctly understand the activity of these neurotransmitters [6,7,9,10]. Turnover is a ratio of metabolite to neurotransmitter concentrations that provides an understanding of the activity of these neurotransmitters within the synapse. Thus, turnover is conversely related to neurotransmitter concentration; as the turnover decreases, there is a higher activity of the neurotransmitter in the synapse. Therefore, an increase in turnover reflects a decrease in synaptic activity of 5-HT and DA. Turnover provides a measure of how neurotransmitters are being utilized to alter cognition or emotional activity, rather than simply measuring stored neurotransmitters that are not active. Investigating the relationship between neurotransmitter turnover and affective state can provide novel neuroendocrine insights and inform the brain–welfare relationship [11]. Ultimately, the brain controls all homeostatic mechanisms, including behavioral, that relate to poultry production and growth [8]. Utilizing physiological measures of hen welfare, such as 5-HT and DA turnover, in addition to visual (or physical) assessments, provides a more robust insight into the hens’ affective state, especially in conventionally caged hens, where outward behavior differences may not be observed.

Thus, the objective of this project was to evaluate the effects of PAWS on the welfare (both physical and physiological) of conventionally caged laying hens. The hens housed under PAWS were hypothesized to have improved welfare compared to hens housed under fluorescent lighting.

## 2. Materials and Methods

All procedures were approved by the Purdue Animal Care and Use Committee (PACUC# 2110002205).

### 2.1. Experimental Design

A commercial laying hen complex that housed flocks under conventional lighting and PAWS provided access for all data and sample collection. Three separate flocks located in the same complex were followed throughout a lay cycle. All flocks consisted of Leghorn white-feathered hens and were housed in conventional cages. Two of the flocks were housed under PAWS, but with different proprietary light recipes (PAWS1 and PAWS2), and the third flock was housed under conventional (fluorescent) lighting (CON). Aside from the lighting system used, all other management parameters were the same across flocks and met the United Egg Producer welfare standards [12]. Both PAWS recipes were formulated with the entirety of the LED light spectra, but due to each recipe’s unique pattern of nanosecond pulses, the lighting in the barn appeared as a red color to the human eye. The pulsing pattern was not visually detectable. The flocks were to be sampled at 22, 32, 52, and 72 weeks of age (woa). However, molt schedules and the 2022 Highly Pathogenic Avian Influenza outbreak reduced sampling from each flock as intended. Exact ages sampled and the associated production phase for analysis, from each flock, are displayed in Table 1.

### 2.2. Welfare Quality Scores

At each timepoint, researchers evaluated the physical welfare of 50 birds per flock using the European Union Welfare Quality^®^ (WQ) Assessment protocol for poultry [13]. Birds were selected randomly across the cage tiers, aisles, and barn length. Welfare parameters measured included (1) deformation of the keel bone, (2) skin lesions, (3) plumage damage, (4) plumage dirtiness, (5) lice/mite infestation, (6) foot pad condition, (7) toe damage, (8) enlarged crops, (9) eye condition, (10) comb wounds and abnormalities, and (11) beak condition and abnormalities. Parameters were scored on either a yes/no basis or a score of 0–2, where 0 was the ideal condition and 2 was the worst condition.

### 2.3. Neurotransmitter Turnover

From each flock, ten birds were randomly selected from those that were evaluated for WQ scores and euthanized via cervical dislocation for brain collection. Brains were extracted within five minutes of euthanasia, immediately placed on dry ice, and stored at −80 °C until analysis. Brains were first hemisected, then the right side was microdissected for biogenic amine analyses into the following brain sections: caudal mesencephalon (CM; caudal raphe), rostral mesencephalon (RM; dorsal raphe and ventral tegmental area), diencephalon (DI; thalamus and hypothalamus), and telencephalon (TEL; including hippocampus and basal nuclei). Each microdissected brain region was weighed and processed for mass spectrometry as reported previously by the Fraley Lab [6,7,9]. Briefly, the brain tissue was homogenized with 10 μL of internal standard (Cayman Chemical, Ann Arbor, MI, USA) and 10 μL of acetonitrile per mg of brain tissue with 0.01% ascorbic acid using 1.0 mm glass beads [14]. Internal standard included heavy hydrogen isotopes at the level of 10 ng/μL for 3-methoxytyramine (3-MT), 3,4-dihydroxyphenylacetic acid (DOPAC), homovanillic acid (HVA), DA, 5-HT, and 5-hydroxyindoleacetic acid (5HIAA). Homogenized samples were vortexed for 10 min and centrifuged for 10 min at 12,000 rpm. Supernatant was removed and placed into a microcentrifuge tube to evaporate inside a speedvac for 24 h. Dried samples were stored at −80 °C until resuspension in 75 μL of 9:1 HILIC A:B [14]. Resuspended samples were vortexed until dried materials were homogenized again, approximately 30 min, then centrifuged at 12,000 rpm for 10 min. Again, supernatant was collected into mass spectrometry tubes and analyzed on an Agilent 6460 Triple Quadrupole Mass Spec (Agilent Technologies, Santa Clara, CA, USA). The results are presented in neurotransmitter concentrations (ng/mg of tissue). These concentrations were applied to turnover equations for 5-HT (5-HT turnover = 5HIAA/5-HT) and DA (DA turnover = [HVA + 3-MT + DOPAC]/DA) [15]. Turnover was calculated to gain a greater understanding of the neurotransmitter activity that cannot be inferred from static levels alone.

### 2.4. Statistics

Welfare quality data were analyzed with R 4.2.2 software [16] with flock as the experimental unit. Since not all treatments were present at all production phases, multiple statistical models were utilized. First, each production phase that contained more than one treatment (peak and post-peak) was analyzed with a separate model for the fixed effect of light. Then, each flock lit with a specific lighting type was independently analyzed for the fixed effect of the production phase. The effect of lighting type or production phase on welfare quality data was determined with the calculation of odds ratios. The odds ratios were performed with Tukey post hoc pairwise comparisons when the chi-square test on the main effect was significant. The odds ratios assessed the odds of a treatment or production phase to be the ideal condition (either a score of “no” or “0”). Differences in serotonin and dopamine turnover were determined with a one-way ANOVA among lighting types within each production phase, followed by Fisher’s LSD post hoc test.

## 3. Results and Discussion

### 3.1. Brain Neurotransmitter Activity and Affective State

No significant or meaningful differences among lighting types were observed in dopamine or associated metabolite levels, nor in dopamine turnover at any age. Excessive DA activity is generally associated with increased aggression in numerous mammalian and avian species [17,18,19]. Dopamine may alter behavior by changing signaling within the brain and can be recognized as a mediator of aggression [17]. The mesolimbic pathway for DA is used to investigate the reward and reinforcement of aggressive behaviors. This pathway can shed light on the relationship between how DA functions to elicit aggression and how defending a resource or performing a behavior can become rewarding and addictive [20], including in poultry [9]. Reducing DA receptor activity with D1 and D2 receptor antagonists is the current treatment to suppress aggressive behavior in humans with psychotic conditions [20]. Highly aggressive strains of laying hens with D1 and D2 receptor antagonists showed decreased feather pecking than hens without the antagonist [21,22]. In the current study, PAWS lighting did not alter DA activity, indicating that the novel light did not increase or decrease aggression compared to the standard-lit hens.

**Figure 1:**
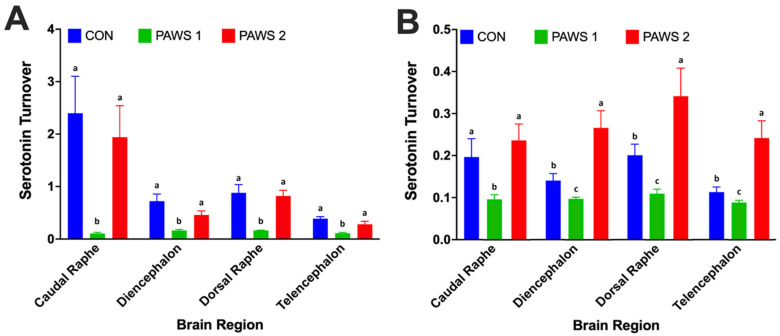
Serotonin turnover in the brain of commercial, conventionally caged White Leghorn hens at (**A**) the peak production phase of CON (32 weeks of age [woa]), PAWS1 (32 woa), and PAWS2 (40 woa); and (**B**) the post-peak production phase of CON (52 woa), PAWS1 (52 woa), and PAWS2 (50 weeks). Differing letters within a brain region indicate statistical differences (*p* < 0.05).

The serotonin turnover of PAWS1 hens was reduced compared to CON and PAWS2 hens at all brain locations in the peak production phase, suggesting increased serotonin synaptic activity in these birds (*p* < 0.05; Figure 1A). At the post-peak production phase, PAWS1 hens had the lowest serotonin turnover, followed by CON hens, while PAWS2 hens had the highest turnover, except at the caudal raphe where there was no difference between CON and PAWS2 (*p* < 0.05; Figure 1B). The reason for the increased serotonin turnover of PAWS2 hens at the post-peak production phase is not clear; there may have been an unknown stressful event that could have influenced brain serotonin activity at the time of collection. Alternatively, the range of serotonin turnover is quite small in the post-peak production phase (Figure 1B), especially compared to the peak phase. Estrogen production begins increasing at the start of egg production, and reaches the highest concentrations around 50 weeks of age [23], the same age as the post-peak production phase sampling in the current study. Additionally, serotonin concentrations have been observed to increase with increases in estrogen levels [24]. Serotonin activity likely increases with age as the estrogen levels continue to increase through post-peak production, resulting in a lower serotonin turnover at the post-peak phase. A decrease in serotonin turnover is related to an increase in synaptic serotonin activity and an improved mood, or affective state [25]. Without enough 5-HT available in the synapse, mammals have been categorized with depressive moods, increased anxiety, and poor welfare [25,26]. Depressive patients are known to have a high 5-HT turnover, and when administered selective serotonin re-uptake inhibitors to treat depressive symptoms, 5-HT turnover significantly decreased, thus increasing 5-HT synaptic activity [25]. Poultry studies have corroborated these findings by demonstrating that decreased serotonin turnover—thus increased synaptic activity—is related to an improved affective state in ducks [6]; conversely, reduced serotonin activity—increased serotonin turnover—is related to poorer affective states [9]. Other studies in poultry have reported that increased 5-HT turnover is associated with increased aggression [27] or environmental stressors [28,29,30,31]. The reduced serotonin turnover in PAWS1 hens compared to CON suggests that this recipe of PAWS light was not perceived as a stressor. The PAWS1 lighting recipe may have had a calming effect on the layers in conventional housing throughout the lay cycle. However, this effect does not seem to be replicated in the hens housed under the PAWS2 lighting recipe, as indicated by the increased serotonin turnover at the peak and post-peak production phases. As serotonin turnover should decrease with age as estrogen production increases [23,24], PAWS2 hens should have had a lower serotonin turnover compared to the other flocks at the peak production phase since they were 8 weeks older at that time. However, PAWS2 had the highest serotonin turnover at both timepoints. Future research to identify optimal PAWS recipes for laying hens would be valuable.

### 3.2. Welfare Quality

Physical welfare quality parameters were scored on a Likert scale of 0–2 (0 being optimal and 2 being the worst); except for comb abnormalities, toe damage, and keel tip fractures, which were scored on a yes/no basis. Since all hens were beak-trimmed, the lowest beak score a hen could receive was 1. Therefore, a beak score of 1 was the reference for odds ratio analysis. Some parameters could not be analyzed as all hens at all timepoints received a perfect score (a score of 0). These included comb abnormalities, comb wounds, foot condition, toe damage, skin lesions, and head feathers. Those parameters are not discussed further as all birds, regardless of age or treatment, did not experience negative welfare due to them. Score frequencies of each welfare quality parameter can be found in Table S1.

All lighting types had similar odds of having a keel tip fracture and a keel score greater than 0 at the peak production phase (*p* > 0.09). Both the CON and PAWS1 hens had worse odds of their keel receiving a score greater than 0 and having a tip fracture at the post-peak production phase compared to the peak phase (*p* < 0.001; Table 2), meaning they were more likely to have a deviated or damaged keel and a tip fracture at the post-peak phase than at the peak phase. On the other hand, the keel bone score and likelihood of tip fracture of PAWS2 hens remained consistent between these phases (*p* > 0.40). This led to the CON flock having 6.17 times and the PAWS1 flock having 4.02 times greater odds of a keel score greater than 0 compared to PAWS2 at the post-peak production phase (*p* < 0.03; Table 3). Both CON and PAWS1 flocks were more likely to have a keel tip fracture compared to PAWS2 (*p* < 0.003; Table 3). However, by the post-molt production phase, PAWS2 hens had greater odds of having a keel tip fracture and keel score greater than 0 compared to the post-peak phase (*p* < 0.02; Table 2).

The keels of all treatments worsened in condition with age, but the PAWS2 hens were able to maintain keel condition until after the post-peak phase, whereas CON and PAWS1 keel condition declined after the pre-peak and peak phases. Additionally, PAWS2 hens were eight weeks older than CON and PAWS1 hens at the peak production phase (40 woa vs. 32 woa, respectively). Most initial keel tip fractures are acquired between 24 and 48 woa, peak egg production, with the highest rate of occurrence from 24 to 32 woa [32]. More keel tip fractures would have been expected in the PAWS2 hens compared to the other flocks at the peak production phase, since they were older; however, that was not the case in the current study. Furthermore, the prevalence of tip fractures remained consistent between the peak and post-peak phases (40 vs. 52 woa) of PAWS2 hens and was lower compared to the CON and PAWS1 hens. The decreased prevalence of keel tip fractures in PAWS2 may indicate that the bone quality of these hens was superior to that of the hens in the CON and PAWS1 flocks. Tibia and humerus breaking strength has been reported to be higher in hens with normal, non-fractured, keel bones compared to hens with deformed keels [33]. Keel tip fractures in aging, caged laying hens are not abnormal and likely the result of bone brittleness from decreased structural bone content and lack of movement [33,34,35]. The keels of PAWS2 hens were able to maintain structural integrity longer than the other lighting types, with most hens succumbing to keel fractures following the post-peak phase instead of prior.

Beak score was not affected by lighting type at the peak production phase (*p* = 0.06), or by production phase in the CON and PAWS2 flocks (*p* > 0.20). However, PAWS1 hens were less likely to have a beak score of 2 compared to PAWS2 (*p* = 0.001) or CON (*p* = 0.03) hens at the post-peak production phase (Table 3). The PAWS1 hens were more likely to have a beak score of 2 at the pre-peak and peak production phases compared to the post-peak production phase (*p* < 0.02; Table 2). Beaks cannot improve in score over time, so this result was likely due to random sampling.

Feather quality was largely influenced by age rather than lighting type. At the peak production phase, the neck and crop feathers of CON and PAWS1 hens were less likely to score greater than 0 compared to PAWS2 hens (*p* < 0.001; Table 3). There was a lighting type effect for back (*p* < 0.001), rump (*p* < 0.001), keel (*p* < 0.001), and belly (*p* < 0.001) feathers at the peak production phase, but pairwise comparisons were not possible since some flocks did not contain scores greater than 0. All CON and PAWS1 hens obtained feather scores of 0 for those body areas, except the rump of CON hens, where 98% of hens had a score of 0 (Table S1). However, 13–32% of PAWS2 hens had back, rump, keel, and belly feather scores greater than 0 (Table S1). The PAWS2 hens were 8 weeks older than the CON and PAWS1 hens at the peak production phase, and increasing age plays a major role in deteriorating feather quality [36,37,38]. Therefore, the age gap better explains the differences in feather quality between the flocks rather than the lighting types.

At the post-peak production phase, the PAW2 flock is just 2 weeks younger than the CON and PAWS1 flocks (50 woa vs. 52 woa), so lighting type effects are indicative of a lighting effect instead of an age × lighting effect. The neck feathers of PAWS2 hens were more likely to have scores of 0 compared to PAWS1 hens (*p* = 0.02), but CON hens were similar in likelihood to both lighting types (*p* > 0.10; Table 3). Crop feathers were impacted by lighting type (*p* = 0.02), with the CON flock tending to be more likely than PAWS2 to have scores of 0 (*p* = 0.07). However, all lighting types had a large percentage of hens with poor crop feather condition (80% or more hens scored greater than 0), likely due to rubbing of the crop area on the feeder [36,37,38].

Pairwise comparisons of back feather scores at the post-peak production phase were not possible, but there was a lighting type effect (*p* < 0.001). All CON hens had back feather scores of 0, but only 70% of PAWS1 hens and 82% of PAWS2 hens had back feather scores of 0 (Table S1). For rump feather condition, CON and PAWS1 hens were more likely to have scores of 0 compared to PAWS2 hens (*p* < 0.006; Table 3). The CON hens were more likely to score a 0 for keel feather condition at the post-peak phase compared to the PAWS1 hens (*p* = 0.04; Table 3), but PAWS2 hens were no more likely than either lighting type. Belly feather condition was not influenced by lighting type at the post-peak production phase (*p* = 0.08).

While there are differences in the feather condition between the lighting types, there is no clear “winner.” Overall, the feather condition is not exceptionally “poor” or “good” in any lighting type, considering the hens were 50–52 woa and housed in conventional cages [36]. The neck feathers of PAWS1 and rump feathers of PAWS2 hens are the only areas with significant differences in proportion of hens scoring greater than 0 compared to the other lighting types. This could be due to random sampling, but could also be indicative of feather pecking. The PAWS2 hens did have an increased serotonin turnover at the post-peak phase, which can be an indicator of stress, and combined with 53% of PAWS2 hens having a rump feather score greater than 0, may indicate the hens under this recipe were pecking rump feathers. However, rump feather loss in conventionally caged hens can be a common location that experiences cage wear of feathers [38].

Age was the driving factor in feather condition scores in the current study. All PAWS1 hens at the pre-peak phase had perfect feathers at every body location (Table S1), so pairwise comparisons within PAWS1 were only possible between the peak and post-peak phases. The neck and crop feathers of CON and PAWS1 hens were more likely to have a score of 0 at the peak production phase compared to the post-peak production phase (*p* < 0.001; Table 2), but there was no difference in odds between those phases for PAWS2 hens. For CON hens, there was a production phase effect for belly feathers (*p* < 0.001), with all hens having a score of 0 at the peak phase versus 80% of hens having a belly feather score of 0 at the post-peak phase (Table S1). However, back feathers, keel feathers, and rump feathers of CON hens were not affected by the production phase (*p* > 0.20). There was an effect of production phase on the back, rump, keel, and belly feather scores for PAWS1 hens (*p* < 0.001), but pairwise comparisons were not possible since all hens had perfect feather scores in the pre-peak and peak production phases (Table S1). However, by the post-peak production phase, only 70–86% of PAWS1 hens had feather scores of 0 for the back, rump, keel, and belly locations (Table S1). This trend of feather condition decreasing with the age of the bird is entirely expected. Wearing of feathers on the cage, feather pecking, stocking density, genetics, and stress can all influence feather deterioration as the bird ages [36,37,38,39].

The feathers of PAWS2 hens increased in quality following molting. Their neck and crop feathers were far more likely to score greater than 0 in the peak and post-peak phases compared to the post-molt phase (*p* < 0.02; Table 2). The post-molt phase tended to have greater odds of keel feathers with scores of 0 compared to the peak production phase (*p* = 0.08), with the main effect of production phase being significant (*p* = 0.02). Production phase did affect the PAWS2 back feather, rump feather, and belly feather scores (*p* < 0.001), but pairwise comparisons were not possible since all hens had perfect feather condition at the post-molt phase (Table S1). LaBrash and Scheideler [36] observed a similar pattern in a survey of commercial laying hen farms where feather cover and condition greatly improved immediately following molt until about 15–25 weeks post-molt, when feather condition started declining again.

## 4. Conclusions

Overall, welfare quality parameters were largely influenced by age in typically expected patterns, but these hens were housed in conventional, battery cages with limited opportunity for movement. There were differences in serotonin turnover in one recipe of PAWS-lit hens that suggest they experienced an improved affective state compared to CON-lit hens. However, due to their restricted space allowance, the hens may not have been able to act on any potential behavior differences that the PAWS lights could be influencing. If this study was repeated in a cage-free system, clearer differences between the welfare quality and behavior of hens under PAWS may present themselves. The novel PAWS lighting may be beneficial to the physical and physiological welfare quality in conventionally caged laying hens and warrants research in cage-free environments.

## Figures and Tables

**Table 1:** Ages and assigned productions phase for analysis that commercial White Leghorn hen flocks housed in conventional cages under various lighting types ^1^ were sampled (X) for neurotransmitter activity and welfare quality.

	Production Phase								
	Pre-Peak		Peak			Post-Peak			Post-Molt
Age (weeks)	22		32	40		50	52		70
CON			X				X		
PAWS1	X		X				X		
PAWS2				X		X			X ^2^

^1^ CON = control (fluorescent) lighting; PAWS1 = pulsed alternating wavelength system (PAWS) recipe #1; PAWS2 = PAWS recipe #2 (light recipes proprietary). ^2^ Flock was molted at 63 weeks of age.

**Table 2:** Pairwise comparisons for the main effect of production phase for welfare quality odds ratios within each lighting type ^1^ in commercial White Leghorn hens housed in conventional cages. Only parameters that could be analyzed with odds ratios and resulted in a significant production phase effect are shown.

		Odds Ratio ^2^	SE	95% CI ^3^	*p*-Value
CON					
Peak vs. Post-Peak					
Keel Score		0.12	0.07	(0.04, 0.38)	<0.001
Keel Tip Fracture		0.04	0.02	(0.01, 0.12)	<0.001
Neck Feathers		0.03	0.03	(0.004, 0.23)	0.001
Crop Feathers		0.06	0.03	(0.02, 0.17)	<0.001
PAWS1					
Pre-Peak vs. Peak					
Beak Score		0.67	0.29	(0.24, 1.85)	0.62
Keel Score		0.28	0.12	(0.10, 0.77)	0.01
Pre-Peak vs. Post-Peak					
Beak Score		9.33	7.35	(1.48, 59.07)	0.01
Keel Score		0.06	0.03	(0.02, 0.22)	<0.001
Peak vs. Post-Peak					
Beak Score		13.94	10.87	(2.24, 86.70)	0.002
Keel Score		0.23	0.12	(0.07, 0.78)	0.01
PAWS2					
Peak vs. Post-Peak					
Keel Score		1.75	0.80	(0.60, 5.12)	0.45
Keel Tip Fracture		0.67	0.29	(0.24, 1.85)	0.62
Neck Feathers		2.45	1.03	(0.91, 6.69)	0.09
Crop Feathers		0.20	0.17	(0.03, 1.38)	0.13
Peak vs. Post-Molt					
Keel Score		0.28	0.17	(0.06, 1.20)	0.10
Keel Tip Fracture		0.10	0.05	(0.03, 0.31)	<0.001
Neck Feathers		63.70	67.07	(5.40, 751.47)	<0.001
Crop Feathers		114.00	93.46	(16.69, 778.7)	<0.001
Post-Peak vs. Post-Molt					
Keel Score		0.16	0.10	(0.04, 0.65)	0.01
Keel Tip Fracture		0.15	0.07	(0.05, 0.45)	<0.001
Neck Feathers		26.03	27.43	(2.20, 307.64)	0.01
Crop Feathers		564.00	575.75	(51.55, 6170)	<0.001

^1^ CON = control (fluorescent) lighting, PAWS1 = pulsed alternating wavelength system (PAWS) recipe #1, PAWS2 = PAWS recipe #2 (light recipes proprietary). ^2^ An odds ratio < 1 means the first listed production phase has reduced odds of having a welfare score > 0. An odds ratio > 1 means the first listed production phase has increased odds of having a welfare score > 0. ^3^ 95% CI = 95% confidence interval. If the value of 1 is not included in the 95% CI, then the odds ratio is significant.

**Table 3:** Pairwise comparisons for the main effect of lighting type ^1^ for welfare quality odds ratios at the peak and post-peak production phases in commercial White Leghorn hens housed in conventional cages. Only parameters that could be analyzed with odds ratios and resulted in a significant lighting type effect are shown.

		Odds Ratio ^2^	SE	95% CI ^3^	*p*-Value
Peak					
CON vs. PAWS1					
Neck Feathers		1.00	1.43	(0.04, 28.47)	1.00
Crop Feathers		1.84	1.03	(0.49, 6.86)	0.52
CON vs. PAWS2					
Neck Feathers		0.02	0.02	(0.001, 0.19)	<0.001
Crop Feathers		0.05	0.03	(0.02, 0.19)	<0.001
PAWS1 vs. PAWS2					
Neck Feathers		0.02	0.02	(0.001, 0.19)	<0.001
Crop Feathers		0.03	0.02	(0.01, 0.12)	<0.001
Post-Peak					
CON vs. PAWS1					
Beak Score		7.58	6.02	(1.18, 48.71)	0.03
Keel Score		1.53	1.04	(0.31, 7.54)	0.80
Keel Tip Fracture		1.21	0.57	(0.40, 3.65)	0.91
Neck Feathers		0.44	0.18	(0.17, 1.16)	0.12
Rump Feathers		0.23	0.16	(0.05, 1.13)	0.08
Keel Feathers		0.07	0.08	(0.01, 0.88)	0.04
CON vs. PAWS2					
Beak Score		0.50	0.22	(0.18, 1.41)	0.26
Keel Score		6.17	3.72	(1.50, 25.34)	0.01
Keel Tip Fracture		5.27	2.38	(1.83, 15.21)	0.001
Neck Feathers		1.36	0.57	(0.52, 3.60)	0.74
Rump Feathers		0.06	0.04	(0.01, 0.27)	<0.001
Keel Feathers		0.23	0.26	(0.02, 3.30)	0.40
PAWS1 vs. PAWS2					
Beak Score		0.07	0.05	(0.01, 0.41)	0.001
Keel Score		4.02	2.13	(1.26, 13.91)	0.02
Keel Tip Fracture		4.34	1.90	(1.56, 12.10)	0.002
Neck Feathers		3.07	1.29	(1.15, 8.19)	0.02
Rump Feathers		0.25	0.11	(0.09, 0.71)	0.005
Keel Feathers		3.17	1.98	(0.74, 13.68)	0.15

^1^ CON = control (fluorescent) lighting, PAWS1 = pulsed alternating wavelength system (PAWS) recipe #1, PAWS2 = PAWS recipe #2 (light recipes proprietary). ^2^ An odds ratio < 1 means the first listed treatment has reduced odds of having a welfare score > 0. An odds ratio > 1 means the first listed treatment has increased odds of having a welfare score > 0. ^3^ 95% CI = 95% confidence interval. If the value of 1 is not included in the 95% CI, then the odds ratio is significant.

## Data Availability

Datasets are not currently published but can be solicited from the corresponding author.

## Supplementary Materials

The following supporting information can be downloaded at: https://www.mdpi.com/article/10.3390/ani16020241/s1, Table S1. Frequency (%) of welfare quality parameter scores for each lighting type at every production phase in commercial White Leghorn hens housed in conventional cages.

## Abbreviations

The following abbreviations are used in this manuscript:

PAWS	Pulsed Alternating Wavelength System
5-HT	Serotonin
DA	Dopamine
woa	Weeks of age
WQ	Welfare quality
CM	Caudal mesencephalon
RM	Rostral mesencephalon
DI	Diencephalon
TEL	Telencephalon
3-MT	3-Methoxytyramine
DOPAC	3,4-Dihydroxyphenylacetic acid
HVA	Homovanillic acid
5HIAA	5-Hydroxyindoleacetic acid
