# Supplementary material for: Evaluation of Pulsed Alternating Wavelength System Lighting on the Welfare Quality and Serotonin Turnover of Commercial Laying Hens Throughout a Lay Cycle
Source: Animals (Basel). 2026 Jan 13;16(2):241. doi: 10.3390/ani16020241 (PMC12838284; doi:10.3390/ani16020241)
Supplement: Supplementary file 1 [file animals-16-00241-s001.zip › animals-4057482-supplementary/DK21-03 WQLayerPAWSSupplementalTable1.pdf]

Supplemental Table 1. Frequency (%) of welfare quality parameter scores[1] for each lighting type[2] at every production phase in commercial White Leghorn hens housed in conventional cages.

	Pre-Peak	Peak			Post-Peak			Post-Molt
	PAWS1	CON	PAWS1	PAWS2	CON	PAWS1	PAWS2	PAWS2
Beak Score								
0	0.0	0.0	0.0	0.0	0.0	0.0	0.0	0.0
1	72.0	83.7	63.3	69.6	76.0	96.0	61.2	66.0
2	28.0	16.3	36.7	30.4	24.0	4.0	38.8	34.0
Keel Score								
0	68.0	42.9	37.5	23.9	8.2	12.0	35.4	8.0
1	28.0	51.0	62.5	73.9	91.8	88.0	58.3	90.0
2	4.0	6.1	0.0	2.2	0.0	0.0	6.3	2.0
Keel Tip Fracture[3]								
0	100.0	87.8	81.3	69.6	22.5	26.0	60.4	18.4
1	0.0	12.2	18.7	30.4	77.5	74.0	39.6	81.6
Head Feathers								
0	100.0	100.0	100.0	100.0	94.0	90.0	89.6	100.0
1	0.0	0.0	0.0	0.0	6.0	8.0	10.4	0.0
2	0.0	0.0	0.0	0.0	0.0	2.0	0.0	0.0
Neck Feathers								
0	100.0	98.0	98.0	43.5	58.0	38.0	65.3	98.0
1	0.0	2.0	2.0	37.0	42.0	60.0	26.5	2.0
2	0.0	0.0	0.0	19.5	0.0	2.0	8.2	0.0
Crop Feathers								
0	100.0	79.6	87.8	17.4	20.0	6.0	4.1	96.0
1	0.0	20.4	12.2	63.0	80.0	94.0	83.7	4.0
2	0.0	0.0	0.0	19.6	0.0	0.0	12.2	0.0
Back Feathers								
0	100.0	100.0	100.0	87.0	100.0	70.0	81.6	100.0
1	0.0	0.0	0.0	8.7	0.0	30.0	14.3	0.0
2	0.0	0.0	0.0	4.3	0.0	0.0	4.1	0.0
Rump Feathers								
0	100.0	98.0	100.0	82.6	94.0	78.0	46.9	100.0
1	0.0	2.0	0.0	4.4	6.0	22.0	49.0	0.0
2	0.0	0.0	0.0	13.0	0.0	0.0	4.1	0.0
Keel Feathers								
0	100.0	100.0	100.0	82.6	98.0	78.0	91.8	98.0
1	0.0	0.0	0.0	15.2	2.0	22.0	8.2	2.0
2	0.0	0.0	0.0	2.2	0.0	0.0	0.0	0.0
Belly Feathers								
0	100.0	100.0	100.0	68.2	80.0	86.0	67.3	100.0
1	0.0	0.0	0.0	13.6	20.0	14.0	24.5	0.0
2	0.0	0.0	0.0	18.2	0.0	0.0	8.2	0.0

[1] Welfare quality scored on a 0-2 scale, where 0 = ideal condition and 2 = worst condition

[2] CON = control (fluorescent) lighting, PAWS1 = pulsed alternating wavelength system (PAWS) recipe #1, PAWS2 = PAWS recipe #2 (light recipes proprietary)

[3] Scored as absence (0) or presence (1)
